# *Letter to the Editor:* Ordering the Chaos in Cannabinoid-Related Research: Is it Time for a Task Force on Taxonomy?

**DOI:** 10.1089/can.2020.0145

**Published:** 2021-04-15

**Authors:** Nicholas H.B. Schräder, Marieke C. Bolling, André P. Wolff

**Affiliations:** ^1^ Department of Dermatology and University Medical Center Groningen, University of Groningen, Groningen, the Netherlands.; ^2^ Department of Anesthesiology Pain Medicine, University Medical Center Groningen, University of Groningen, Groningen, the Netherlands.

To the editor of *Cannabis and Cannabinoid Research*, the cannabinoid research associations and institutes, and to the scientific community endeavoring the field of cannabinoid-related research:

An increasing number of countries are loosening restrictions on both recreational and medical cannabinoid use, which has inevitably led to growing scientific interest. In fact, cannabinoid research is one of the fastest growing scientific fields, seeing exponential number of publications per year on topics spanning therapeutic, preventive, and basic research in numerous diseases.^1^

As a result of expanding research, there is an increasing heterogeneity of terminology in this immense data set. One can get lost in the numerous terms describing cannabinoid-related substances, their chemical constituents, and biological derivatives. We can already see the natural evolution of scientifically appropriated cannabinoid-related terminology moving from ordered to chaotic, where a simple literature search must encompass multiple terms, such as *cannabis, cannabinoids, delta-9-tetrahydrocannabinol, THC, cannabidiol, CBD, marijuana, nabilone, dronabinol, and nabiximols*, taken from the search query of a recent meta-analysis.

The problem with heterogeneous terminology for cannabinoid-related science is that it facilitates confusion among both new and established scientists. In the absence of a framework, researchers are likely to harness imprecise nomenclature used within their network, leading to the fragmentation of fields of thought. Without uniform communication, authors are obliged to develop their own classifications whereby research studies cannot reliably be compared. This is a significant hurdle to the scientific community who wish to communicate sound and reproducible research, and international organizations such as the United Nations, who are impaired in their decision making “…by a lack of international standards on issues such as terminology,” with respect to cannabinoid-related policy.^2^ It must go without saying that harnessing the benefits from a terminological consensus will excel the efficiency in cannabinoid-related scientific research (from basic science through clinical trials and meta-analyses), streamline collaboration, and maintain coherent education.

Of recent example, we highlight evolving terminology from the *sativa-indica* debate in which research concluded that the ubiquitous interbreeding and hybridization of these rendered their distinctions meaningless. The fruit of this debate resulted in a well-founded incorporation and use of the term *chemovar,* providing a more accurate description of cannabis' chemical variety.^3^ Despite the importance of this implication, it may yet remain dormant to much of the scientific community until a formal introduction is made.

The lack of a taxonomy consensus is not unique to cannabinoid-related science, and has frequently been addressed in a number of scientific fields. Recent history has shown us that cooperative consolidation of nomenclature, based on scientific consensus, leads to improvement and efficiency of patient-related health care, and remains an ongoing process.^4^ We can also take example from The International Association for the Study of Pain that, when presented with increasingly heterogeneous nomenclature, created a “Task Force on Taxonomy,” laying the foundation to a consensus on international standards for the diagnosis of chronic pain conditions. Furthermore, catalyzed by increasing complexity and often contradictory definitions for cardiomyopathy, the American Heart Association formulated a rigorous classification system to centralize further dividing schools of thought.

The unmet need for a taxonomy consensus in the field of cannabinoid research is no fault of the scientists, but is due to the intricate nature of this vastly expanding field. As we endeavor and discover more, cannabinoid-related terminology will change, terms become futile, and definitions not up to date. A taxonomy for cannabinoid-related science should be dynamic, allowing for adaptations when new research tells us otherwise. Cannabinoid-related scientific terminology should be overseen and systematically regulated. It should include the expert opinion and management of already established organizations that have addressed the ambiguity of nomenclature for the innumerous cultigens.

At the core of this field of research are the scientific societies, the International Cannabinoid Research Society (ICRS) and International Association for Cannabinoid Medicines (IACM), which must take the first step in a taxonomy consensus. Irrespective of whether you as a reader accept this proposal, let this be a cornerstone to finding order in the chaos.

We, therefore, would like to encourage the experts in cannabinoid-related science and the wider scientific community to endeavor the formation of a task force on taxonomy that can be used on all levels of scientific research and education. To develop a classification system that is pragmatic and all-encompassing, yet precise. One that can be looked up to in the worldwide scientific community.

Sincerely,

A fellow clinician and researcher, Nicholas H.B. Schräder, MD.

## Abbreviations Used

CBD: cannabidiol
IACM: International Association for Cannabinoid Medicines
ICRS: International Cannabinoid Research Society
THC: tetrahydrocannabinol
